# Supplementary material for: Novel Driver Strength Index highlights important cancer genes in TCGA PanCanAtlas patients
Source: PeerJ. 2022 Aug 11;10:e13860. doi: 10.7717/peerj.13860 (PMC9375969; doi:10.7717/peerj.13860)
Supplement: Supplemental Information 2 [file peerj-10-13860-s002.zip › COHORTS/genes plots/stage/2021_8_16_14_9_distribution_stage_II.pdf]

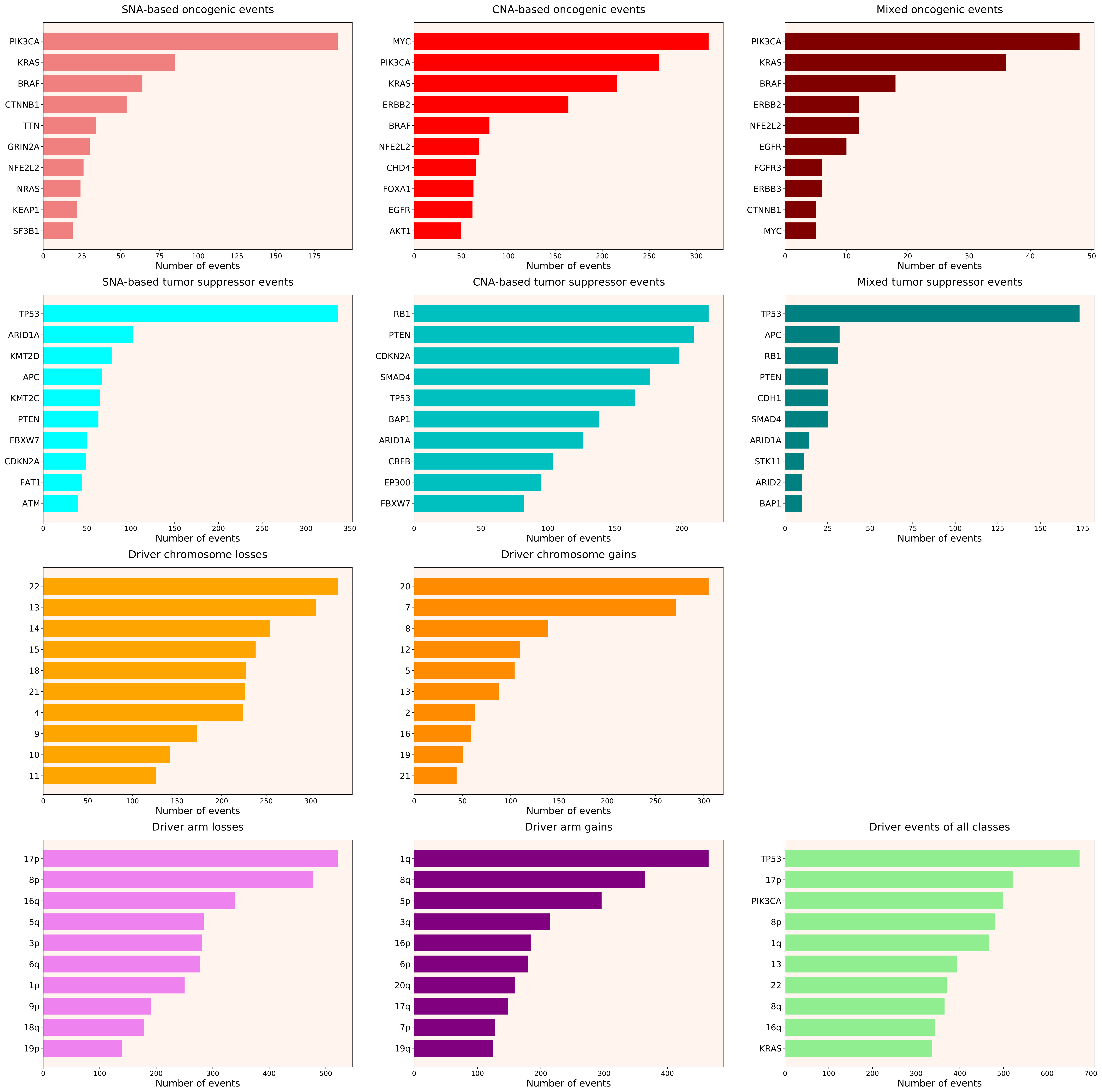
SNA-based oncogenic events
PIK3CA
KRAS
BRAF
CTNNB1
TTN
GRIN2A
NFE2L2
NRAS
KEAP1
SF3B1
0
25
50
75
100
125
150
175
Number of events
CNA-based oncogenic events
MYC
PIK3CA
KRAS
ERBB2
BRAF
NFE2L2
CHD4
FOXA1
EGFR
AKT1
0
50
100
150
200
250
300
Number of events
Mixed oncogenic events
PIK3CA
KRAS
BRAF
ERBB2
NFE2L2
EGFR
FGFR3
ERBB3
CTNNB1
MYC
0
10
20
30
40
50
Number of events
SNA-based tumor suppressor events
TP53
ARID1A
KMT2D
APC
KMT2C
PTEN
FBXW7
CDKN2A
FAT1
ATM
0
50
100
150
200
250
300
350
Number of events
CNA-based tumor suppressor events
RB1
PTEN
CDKN2A
SMAD4
TP53
BAP1
ARID1A
CBFB
EP300
FBXW7
0
50
100
150
200
Number of events
Mixed tumor suppressor events
TP53
APC
RB1
PTEN
CDH1
SMAD4
ARID1A
STK11
ARID2
BAP1
0
25
50
75
100
125
150
175
Number of events
Driver chromosome losses
22
13
14
15
18
21
4
9
10
11
0
50
100
150
200
250
300
Number of events
Driver chromosome gains
20
7
8
12
5
13
2
16
19
21
0
50
100
150
200
250
300
Number of events
Driver arm losses
17p
8p
16q
5q
3p
6q
1p
9p
18q
19p
0
100
200
300
400
500
Number of events
Driver arm gains
1q
8q
5p
3q
16p
6p
20q
17q
7p
19q
0
100
200
300
400
Number of events
Driver events of all classes
TP53
17p
PIK3CA
8p
1q
13
22
8q
16q
KRAS
0
100
200
300
400
500
600
700
Number of events
